# Uncovering molecular markers of the microvascular endothelial response in sepsis-associated acute kidney injury: a translational study in mice and humans

**DOI:** 10.1186/s40635-025-00801-4

**Published:** 2025-09-02

**Authors:** T. J. van der Aart, G. Molema, R. M. Jongman, H. R. Bouma, J. Koeze, J. Moser, M. van Londen, M. Hackl, A. B. Diendorfer, J. C. ter Maaten, M. van Meurs, M. Luxen

**Affiliations:** 1 Department of Internal Medicine, University Medical Center Groningen, University of Groningen, P.O. Box 30.001, 9700 RB Groningen, The Netherlands; 2 Department of Acute Care, University Medical Center Groningen, University of Groningen, Groningen, The Netherlands; 3 Department of Pathology and Medical Biology, Medical Biology section, University Medical Center Groningen, University of Groningen, Groningen, The Netherlands; 4 Department of Anaesthesiology, University Medical Center Groningen, University of Groningen, Groningen, The Netherlands; 5 Department of Clinical Pharmacy and Pharmacology, University Medical Center Groningen, University of Groningen, Groningen, The Netherlands; 6 Department of Critical Care, University Medical Center Groningen, University of Groningen, Groningen, The Netherlands; 7 Healics study group, Department of Critical Care, University Medical Center Groningen, University of Groningen, Groningen, The Netherlands; 8 Department of Internal Medicine, Division of Nephrology, University Medical Center Groningen, University of Groningen, Groningen, The Netherlands; 9 TAmiRNA GmbH, Wien, Austria

**Keywords:** Acute kidney injury, Early diagnostics, Endothelial activation, Endothelial cells, Emergency department, Intensive care unit, SA-AKI, Sepsis

## Abstract

**Introduction:**

Endothelial cells play a central role in the pathophysiology of sepsis-associated acute kidney injury (SA-AKI), yet we have limited understanding of the markers of microvascular-specific response. We therefore employed a translational approach integrating spatially resolved transcriptomics in a mouse SA-AKI model with validation in human kidney tissues and plasma, aiming to define the molecular signature of the endothelial response to SA-AKI in mice and in human patients.

**Methods:**

In this post hoc analysis of prospectively collected data, we identified sepsis-associated target mRNAs and validated their expression via RT-qPCR in distinct renal microvascular compartments isolated by laser microdissection (LMD) from both cecal ligation and puncture (CLP) mice and post-mortem kidney biopsies of SA-AKI patients. Additionally, we measured the corresponding circulating proteins in plasma from two patient cohorts with sepsis and SA-AKI: one consisting of patients presenting to the emergency department, and the other of patients with severe sepsis requiring organ support in the ICU.

**Results:**

We identified several differentially expressed genes in the renal microvasculature following sepsis, including *Mt1*, *Mt2*, *Saa3, Hp*, *C3*, *Sparc*, *Mmp8*, and *Chil3*. Whole-organ samples from CLP mice also showed increased expression in the liver and lung. Except for *SPARC*, all genes were similarly upregulated in human kidney biopsies from SA-AKI patients. Circulating protein levels were elevated in sepsis and SA-AKI patients compared to controls; however, only CHI3L1 and MMP8 showed significantly higher levels in SA-AKI versus sepsis across both early and advanced stages.

**Conclusion:**

Our findings reveal markers of the microvascular response to sepsis, which include increased levels of *HP, C3, Chil3/CHI3L1,* and *MMP8*, both at the transcriptomic level in mouse and human kidney microvasculature and at the protein level in circulating plasma of SA-AKI patients. The upregulation of these markers was shared across multiple organs and may reflect widespread endothelial activation contributing to sepsis pathophysiology.

**Supplementary Information:**

The online version contains supplementary material available at 10.1186/s40635-025-00801-4.

## Introduction

Sepsis is a life-threatening host response to an infection often complicated by acute kidney injury, also known as sepsis-associated acute kidney injury (SA-AKI). The occurrence of SA-AKI is strongly associated with increased morbidity and mortality [1–3]. Despite considerable heterogeneity in the molecular processes underlying SA-AKI, current management strategies remain generalized, and no effective personalized treatments are available [4]. The complex pathophysiology of SA-AKI involves multiple interacting mechanisms, including inflammation, mitochondrial damage, tubular epithelial injury, and (micro)vascular dysfunction [5]. Endothelial cells (EC), which form the inner lining of the renal microvasculature, play a central role in the pathophysiology of sepsis upon activation by contributing to disturbed vasomotor regulation, increased vascular permeability, and the recruitment of leukocytes [6, 7]. Although a precise definition of endothelial dysfunction in sepsis is still lacking, there is general consensus that these processes represent its core features [8, 9]. There has been a growing interest in sub-phenotyping sepsis to develop more targeted therapeutic approaches that account for individual variability in pathophysiology. Despite being a central feature in the pathophysiology of SA-AKI, endothelial dysfunction remains poorly characterized, and no therapies to date specifically target endothelial dysfunction. Established vascular markers like angiopoietin-2 and VCAM-1 are limited in capturing endothelial dysfunction in SA-AKI and were underrepresented or non-discriminative in sub-phenotyping efforts [10–14]. A deeper understanding of the molecular and cellular components underlying the microvascular response in SA-AKI may offer mechanistic insights into endothelial activation, thereby facilitating sub-phenotyping, clarifying disease mechanisms, and identifying novel therapeutic targets.

Identifying molecular markers of the microvascular response requires biologically informed approaches and translational validation in human tissues. Traditional in vitro models are limited, as endothelial cells (ECs) rapidly lose their tissue-specific characteristics in culture [15], underscoring the importance of studying EC responses in vivo. Moreover, histopathological evidence shows that endothelial responses to sepsis vary across distinct renal compartments [16], highlighting the need for spatially resolved analysis. Therefore, the present study provides translational validation of candidate sepsis-associated endothelial mRNAs by quantifying their expression via RT-qPCR in renal compartments from both CLP mice and post-mortem kidney biopsies of SA-AKI patients. Additionally, we assess the circulating protein levels of these markers in human patients, thereby bridging molecular findings from animal models to clinical relevance to provide a comprehensive, translational understanding of sepsis-related kidney injury.

## Methods

### Study design

This study is a post hoc analysis of prospectively collected data combining experimental cecal ligation and puncture (CLP) mouse model of SA-AKI, post-mortem human kidney biopsy data, and plasma data from living patients in a cohort study. This study is embedded in the Acutelines data-biobank (www.acutelines.nl) registered in Clinicaltrials.gov as NCT04615065, the Biobank Intensive Care Groningen (BICG) and HEALICS registered in Clinicaltrials.gov as NCT04502511 [17, 18]. Both initiatives aim to advance understanding of sepsis and acute illness pathophysiology and improve patient care.

### Selection mRNA targets

In a previous study using a CLP-sepsis mouse model, we employed RNA sequencing to identify a complete mRNA expression profile across distinct renal microvascular compartments: arterioles, glomeruli, peritubular capillaries, and postcapillary venules [19]. These previously published datasets (Supplemental file 1, S. Table 1-3) informed the current study’s selection of mRNA targets. All data presented in the current manuscript were newly generated and represent original experimental work designed specifically for this study. In this study, we selected mRNAs with increased expression in multiple microvascular compartments and large effect sizes in response to CLP-sepsis. This led to the selection of *Mt1*, *Mt2*, *Saa3*, *Hp*, and *C3* (Supplemental file 1, S. Table 1-3). As glomeruli exhibited the highest number of differentially expressed genes compared to other microvascular compartments (Supplemental file 1, S. Table 1-3), we also included genes specifically upregulated in the glomeruli: *Chil3*, *Sparc*, and *Mmp8*.

### CLP mouse sepsis model

Sepsis was induced through CLP in 8- to 12-week-old C57BL/6 OlaHsd male mice (Envigo, Horst, The Netherlands). Mice were randomly assigned to different groups: untreated control mice (*n* = 8), sham-operated mice (*n* = 10), and CLP mice (*n* = 13). Sham-operated and CLP mice were killed at 4, 7, 24, or 72 h after surgery, as previously described [19]. Experimental procedures were approved and performed in accordance with the University of Groningen Ethical Committee and National and European Guidelines for Animal Care and Use (#8116, AVD1050020184904).

### Human kidney biopsies

Human kidney cortex biopsies were obtained post-mortem from patients with SA-AKI at the ICU of the University Medical Center Groningen (UMCG) as originally described by Aslan et al. [16]. Biopsies were obtained from the renal cortex, and nonseptic kidney biopsies were obtained from six patients with renal cell carcinoma. A healthy section of the cortex was obtained following surgical examination after nephrectomy. All sections were immediately snap-frozen on liquid nitrogen and stored at −80 °C until further analysis. The Medical Ethics Review Committee (METC) of the UMCG reviewed and waived the need for ethics approval for this study (METc 2011/372).

### Human subjects

All patients admitted to the ED from February 2021 until February 2023 from the Acutelines cohort were screened for participation. Acutelines is a multi-disciplinary prospective hospital-based cohort study at the ED of the UMCG. Acutelines’ complete protocol and overview of the current, full data dictionary is available via www.acutelines.nl [18].

Furthermore, we investigated an ICU cohort consisting of severely ill patients with sepsis and SA-AKI necessitating organ support therapy. Patients included in Biobank IC and admitted to the ICU between December 2021 and March 2023 with severe sepsis were screened for inclusion. Of note, within the Dutch healthcare system, ICU admission is reserved for severe illnesses requiring organ support. Sepsis cases not requiring invasive respiratory support, vasopressors, or renal replacement therapy (RRT) are treated in general wards.

Data for this study are collected and managed using REDCap (Vanderbilt University, Nashville, USA). All clinical information, including vital signs and laboratory results, is extracted from the hospital’s electronic health record system (EPIC Systems, USA). Informed consent was obtained from all participants or, when necessary, from legal proxies. All procedures adhered to the principles of the Declaration of Helsinki.

### Human sample collection

Blood samples from the ED cohort were collected upon arrival in EDTA Sarstedt tubes (BD Vacutainer^®^ PPT^TM^, Franklin Lakes, USA). Samples were centrifuged within four hours at 20°C (1500 g, 15 min) and stored at –80°C, undergoing only a single freeze-thaw cycle. Similarly, ICU blood samples were collected within three hours of admission in the same tubes, centrifuged at 4°C (380 g, 15 min), and stored at –80°C.

### Definitions

In the ED cohort, sepsis was diagnosed using the sepsis-3 criteria [20]. The presence and site of infection were post hoc determined by an expert adjudication committee as part of Acutelines, based on the Centers for Disease Control and Prevention consensus definitions [21]. AKI was determined by the Kidney Disease: Improving Global Outcomes (KDIGO) criteria: an increase in plasma creatinine of 1.5 times baseline or an increase in plasma creatinine with ≥26.5 µmol/L [22, 23]. Baseline creatinine was determined by the mean creatinine value up to one year prior to ED presentation when at least three values were available. Urine volume was not considered in AKI diagnosis. All patients underwent an adequate diagnostic trajectory to evaluate other causes of AKI (*e.g.*, post-renal, medication-related, glomerulonephritis) before concluding that AKI was sepsis-associated.

In accordance with the Dutch National Intensive Care Evaluation (NICE) guidelines, sepsis in the ICU cohort was defined as an infection requiring ICU admission for organ supportive therapy [24]. Infection was confirmed by either positive culture results and/or radiological imaging. AKI was determined through the NICE guidelines: the necessity of renal replacement therapy (RRT) within the first 24 h of ICU admission or a plasma creatinine level of ≥300 µmol/L in the past 24 h accompanied by oliguria. Oliguria was defined as a urine output of less than or equal to 150 ml over eight consecutive hours without other causes.

### Acquisition of renal microvascular compartments

To obtain the renal microvascular compartments, specifically arterioles, glomeruli, peritubular capillaries, and postcapillary venules, we used the previously collected laser microdissected (LMD) samples from cryosections of snap-frozen kidneys from the CLP mice [19], as well as from the renal biopsies of patients with SA-AKI as described previously [25].

### Measuring gene transcription levels via RT-qPCR

RNA from the laser microdissected renal microvascular compartments and whole organs of CLP mice and human kidney biopsies were isolated and reverse transcribed as described previously [19]. qPCR was performed using Assay-on-Demand FAM MGB-labeled hydrolysis probes on a ViiA 7 Real-Time PCR System, and the data were analyzed using QuantStudio Real-Time PCR Software (v. 1.3, all from Applied Biosystems, Waltham, MA, USA). Obtained C_q_ values were normalized to the mean of the reference genes *Gapdh* and *Ppia* for mouse samples and normalized to the geometric mean (GeoMean) of *GAPDH*, *PPIA*, and *YWHAZ* for human samples. mRNA transcription levels relative to reference genes were calculated by 2^−ΔCq^.

### Statistical analysis

Statistical analyses were performed, and graphs were created in GraphPad Prism (v. 9.5.0, GraphPad Software, San Diego, CA, USA). We analyzed data using one-way ANOVA with Šidák’s multiple comparisons test. When assumptions were violated, we applied Welch’s ANOVA followed by a post hoc Dunnett’s test, or a Kruskal–Wallis test with Dunn’s post hoc test, as appropriate. For comparisons between two groups, we used an unpaired two-tailed *t*-test or the Mann–Whitney U test, depending on data distribution. A minimum of three observations per group was required to allow for statistical testing. Statistical significance was determined at *p* < 0.05 throughout.

## Results

### Selected mRNA targets are differentially expressed in the renal microvasculature of CLP-sepsis mice

**Fig. 1:**
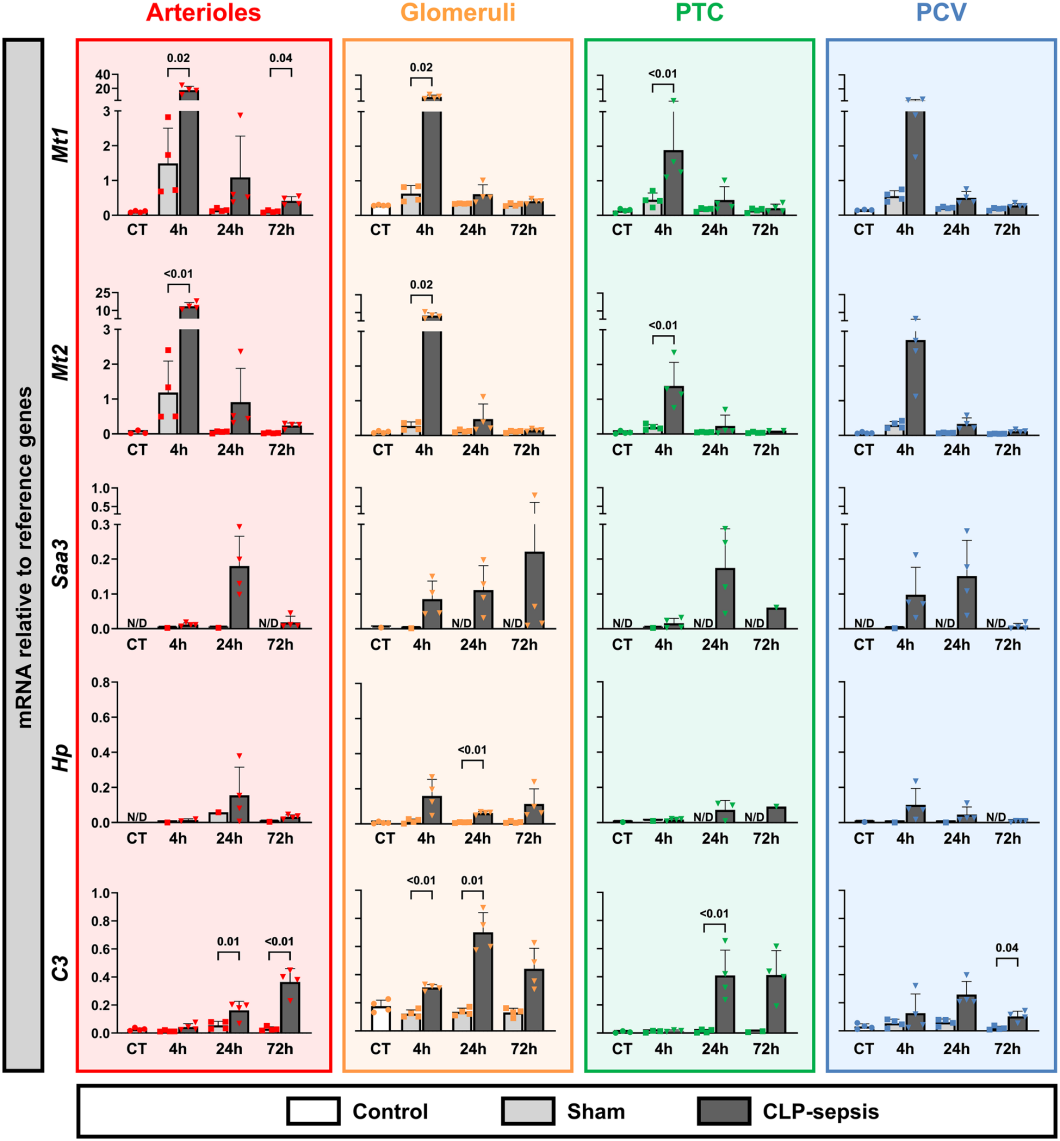

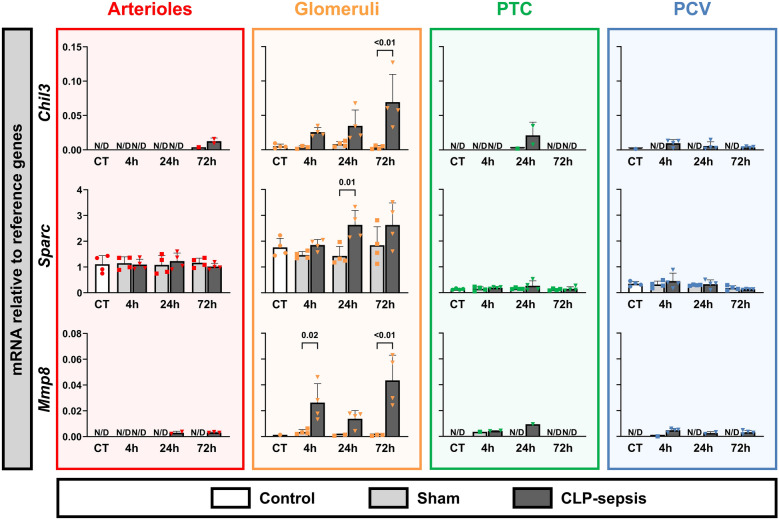
Altered mRNA transcription levels of selected genes in microvascular compartments from CLP-induced sepsis in mice. Gene expression levels of *Mt1*, *Mt2*, *Saa3*, *Hp*, *C3*, *Chil3*, *Sparc*, and *Mmp8* were assessed in various microvascular compartments, including arterioles, glomeruli, peritubular capillaries (PTC) and postcapillary venules (PCV). Microvascular compartments were laser microdissected from mice with CLP-induced sepsis, sham, and controls. Expression was measured relative to reference genes. Data are presented as mRNA levels. Graphs display column means with SD or median with IQR when applicable. Statistical testing was performed using ANOVA or Welch’s ANOVA with post hoc tests, or Kruskal–Wallis (KW) with post hoc Mann–Whitney U tests where appropriate and when at least three datapoints were obtained, and statistical data are shown when *p* < 0.05

Known renal injury markers, such as markers *Lcn2* (NGAL), *Havcr1* (KIM1), *Timp2*, *Igfbp7*, are tubular injury markers and therefore are not indicative of the microvascular response in mouse CLP-sepsis (Supplemental Figure 1). Therefore, based on prior RNA sequencing data (Supplemental file 1) [19], we selected mRNAs with increased expression across multiple renal microvascular compartments and large effect sizes in response to CLP-sepsis. This led to the selection of *Mt1*, *Mt2*, *Saa3*, *Hp*, *C3, Chil3*, *Sparc*, and *Mmp8* (Supplemental file 1, S. Table 1-3). RT-qPCR analysis confirmed significant upregulation of *Mt1*, *Mt2*, *Saa3*, and *C3* across all microvascular compartments following sepsis (Fig. 1). *Mt1* and *Mt2* exhibited early upregulation at 4 h post-CLP, while *C3* and *Saa3* were predominantly upregulated after 24 h. *Chil3* and *Mmp8* exhibited distinct and sustained upregulation within the glomerular compartment, showing early increases that persisted for at least 72 h. *Sparc* mRNA levels were induced in glomeruli at 24 h post-CLP, yet the fold change was lower compared to *Chil3* and *Mmp8*.

### Upregulation of mRNA targets in CLP-sepsis occurs in multiple organs

**Fig. 2:**
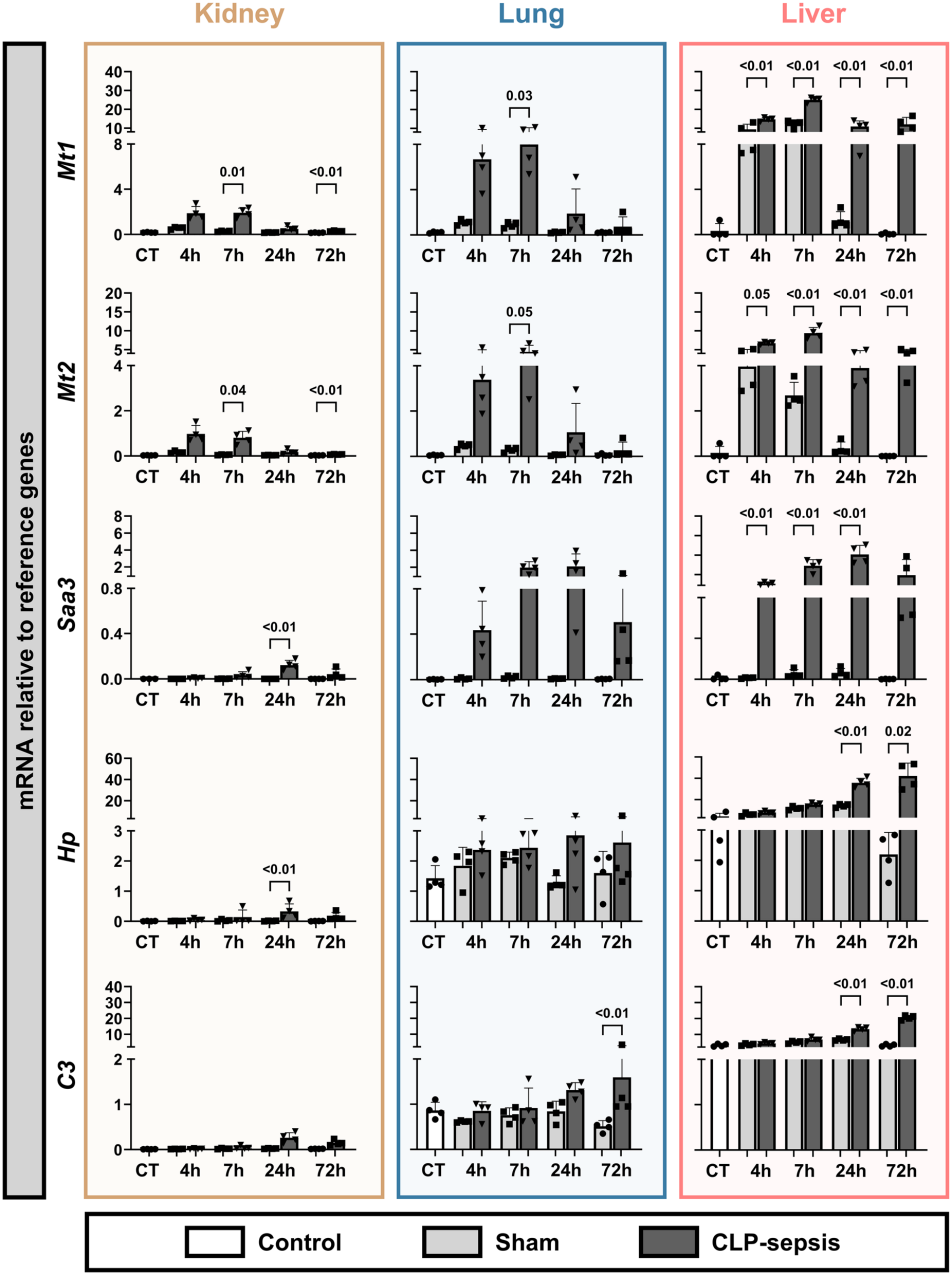

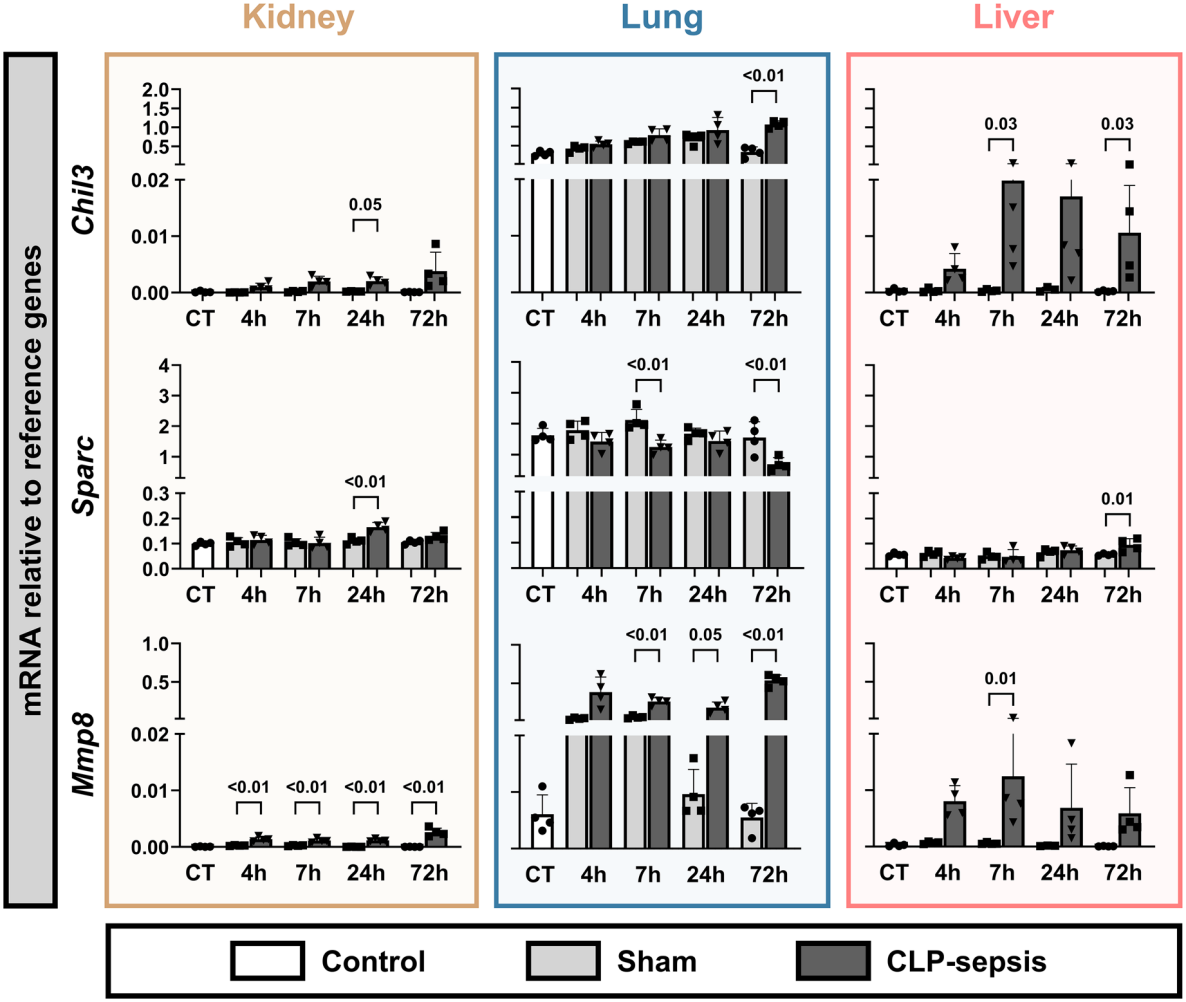
mRNA expression of selected genes in whole kidney, lung, and liver tissue. Gene expression levels of *Mt1, Mt2, Saa3, Hp, C3, Chil3, Sparc,* and *Mmp8* are shown in whole kidney (gold), lung (blue), and liver (pink) tissue, expressed relative to reference genes. Graphs display column means with SD or median with IQR when applicable. Statistical testing was performed using ANOVA or Welch’s ANOVA with post hoc tests, or Kruskal–Wallis (KW) with post hoc Mann–Whitney U tests where appropriate and when at least three datapoints were obtained, and statistical data are shown when *p* < 0.05

To determine whether the observed expression patterns were specific to kidney injury in sepsis, we also examined the selected genes from CLP mice in lung and liver tissues. This revealed that the sepsis-induced expression of all genes was not exclusive to the kidney, with relative expression in the kidney being minor compared to the lungs and liver (Fig. 2). Additionally, we observed organ-specific kinetics in sepsis-induced gene expression. For instance, *Mt1* and *Mt2* were strongly induced in both the kidney and lungs up to 7 hours post-CLP, followed by a rapid decline in expression. In the liver, however, *Mt1* and *Mt2* gene expression remained elevated up to 72 hours after CLP. *Mmp8* and *Chil3* showed clear upregulation in both the kidney and liver following sepsis. In the lungs, both sham injury and sepsis induced *Mmp8*. However, in the sham mice, the induction resolves over time but stays induced for at least 72 h in CLP mice. Changes in *Chil3* expression were less pronounced due to high base level expression. Taken together, these findings demonstrated that the selected genes showed altered expression in response to sepsis in multiple organs and were not restricted to the kidney.

### Gene expression patterns are similar between mice and post-mortem SA-AKI patients

**Fig. 3:**
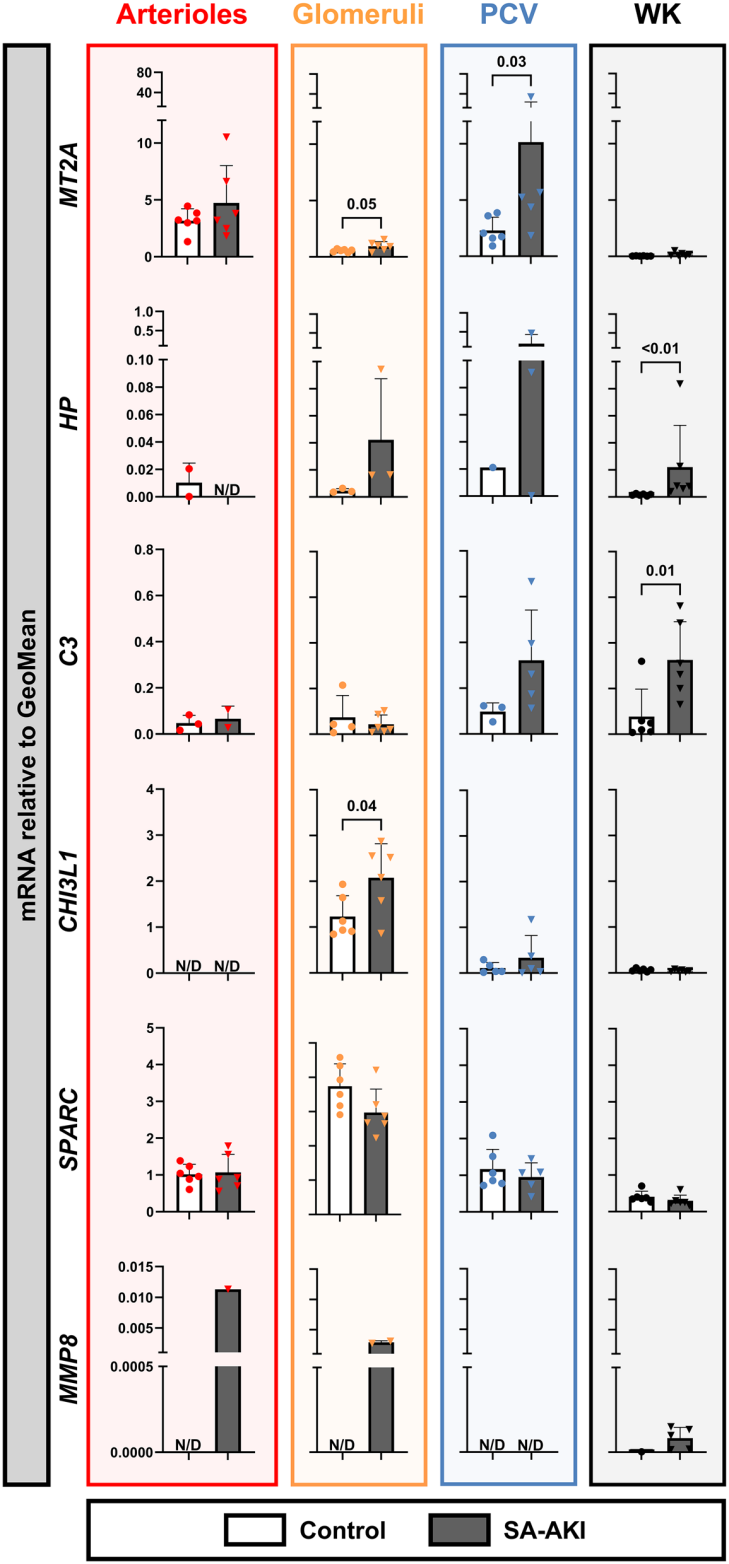
Altered mRNA transcription levels of selected genes in microvascular compartments from patients with sepsis-associated acute kidney injury. Gene expression levels of *MT2A*, *HP*, C3, *CHI3L1*, *SPARC*, and *MMP8* were assessed in various microvascular compartments, including arterioles, glomeruli, postcapillary venules (PCV), and whole kidney (WK) samples. Microvascular compartments were laser microdissected from healthy kidney biopsy tissue (control) and post-mortem kidney biopsies from patients with sepsis-associated acute kidney injury (SA-AKI). Expression was measured relative to the geometric mean (GeoMean) of reference genes. Data are presented as mRNA expression levels. Graphs display column means with SD or median with IQR when applicable. Statistical testing was performed using an unpaired two-tailed t test or Mann–Whitney U test when at least three datapoints were obtained. Statistical data are shown when *p* < 0.05. Of note: the absence of the PTC column is due to mRNA degradation. This issue did not affect the other compartments, as a different staining method is used for their visualization in LMD, which provides protection for the mRNA.

To determine if the microvascular expression patterns observed in mice were similar in humans, we assessed the expression of the selected genes in post-mortem kidney biopsies from patients with SA-AKI and compared these with the microvascular expression levels in healthy kidney tissue (Fig. 3). Human kidney cortex biopsies were obtained within 33 minutes (range 24–150) post-mortem from patients with SA-AKI. *Mt1* and *Saa3* were excluded from this analysis due to the absence of homologous genes in humans. *CHI3L1* exhibited increased expression in glomeruli following SA-AKI, consistent with the induced expression of *Chil3* in mouse glomeruli. Similarly, *MT2A*, *HP*, and *MMP8* mRNA levels were higher across multiple microvascular compartments in SA-AKI patients compared to controls, though statistical significance was not always assessed due to limited data points. *C3* mRNA levels were higher only in whole kidney SA-AKI biopsies compared to controls, but not in the individual microvascular compartments. *SPARC* mRNA levels were similar in both control and SA-AKI biopsies.

### Translating renal mRNA markers to circulating protein markers in patients with SA-AKI and sepsis

Given the limited feasibility of performing kidney biopsies in clinical practice, we next explored whether the corresponding proteins of the identified genes of interest could be detected in circulating plasma from patients with sepsis and SA-AKI. We translated the tissue-based mRNA signals into measurable, cell-free plasma counterparts, resulting in the following detectable proteins: HP, C3a (*C3*), CHI3L1 (*Chil3* in mice), and MMP8. We investigated the plasma levels of these proteins in two patient cohorts: an ED cohort representing the early stage of the clinical trajectory at hospital presentation, and an ICU cohort reflecting a more advanced disease stage requiring organ supportive therapy (Table 1).

**Table 1:** Baseline characteristics of the emergency department and intensive care cohorts (n = 240)

Cohort	Emergency department				Intensive care unit		
	No infection (*N* = 65)	Sepsis (*N* = 67)	SA-AKI (*N* = 61)	*P* value	Sepsis (*N* = 18)	SA-AKI (*N* = 29)	P value
Age Sex (F)	61 [47–70] 32 (49)	67 [53–75] 32 (48)	69 [53–77] 31 (51)	**0.02** 0.94	60 [52–67] 8 (44)	68 [59–71] 14 (48)	0.07 0.87
NEWS2 score SOFA score APACHE IV score	2 [0–4] 1 [0–4]	7 [4-9] 5 [4–6]	5 [3–9] 7 [5–9]	**<0.01** **<0.01**	92 [49–102]	68 [48–117]	0.65
AKI G1 AKI G2/G3 CKD		1 (2)	32 (52) 29 (48) 12 (20)		0 (0)	4 (13.8)	
Baseline creatinine Creatinine μmol/L Urea mmol/L eGFR ml/min/1.73m^2^ Hemoglobin mmol/L Thrombocytes x10^9^/L Leukocytes x10^9^/L CRP mg/L Lactate mmol/L	75 [58–85] 75 [65–83] 5.4 [4.3–7.0] 88 [78–102] 8.1 (1.6) 243 [205–302] 7.9 [6.6–11.0] 19 [6–40] 1.7 [1.0–2.2]	70 [58–85] 73 [55–92] 5.8 [4.2–7.4] 87 [64–102] 8.0 (1.3) 208 [140–264] 11.7 [7.0–15.5] 90 [39–175] 1.4 [1.0–2.3]	75 [65–101] 133 [110–181] 12.5 [7.3–17.1] 41 [26–54] 7.3 (1.3) 217 [151–286] 12.4 [7.2–18.7] 167 [80–257] 2.0 [1.4–3.5]	0.29 **<0.01** **<0.01** **<0.01** **<0.01** **0.03** **<0.01** **<0.01** **<0.01**	178 [88–310] 11.2 [6.1–14.4] 94 [73–102] 7.1 (1.5) 210 [89–262] 8.9 [4.4–17.8] 161 [129–320] 1.6 [1.3–2.2]	150 [90–211] 9.2 [6.6–13.4] 30 [20–48] 6.5 (1.6) 166 [78–265] 12.4 [4.9–19.3] 170 [70–320] 2.0 [1.2–4.1]	0.47 0.89 **<0.01** 0.31 0.67 0.65 0.79 0.41
*Comorbidities* Diabetes CVD Malignancy COPD	5 (8) 7 (10.8) 3 (4.6) 1 (2)	9 (13) 16 (24.2) 2 (3.0) 24 (36)	20 (33) 20 (30.3) 26 (42) 13 (21)	**<0.01** **0.03** **<0.01** **<0.01**	3 (17) 1 (6) 4 (22) 1 (6)	5 (17) 3 (10) 4 (14) 3 (10)	0.96 0.57 0.46 0.57
*Diagnosis admission* Infection/sepsis Cardiac Neurological Respiratory Intoxication Gastro-intestinal Anaphylaxis Other	0 (0) 9 (14) 18 (28) 5 (7) 5 (7) 12 (19) 5 (8) 11 (17)	67 (100)	61 (100)		13 (72) 1 (6)^a^ 1 (6)^b^ 3 (6)^c^	29 (100)	
*Outcome* Length of hospital stay ICU admission Length of ICU stay In-hospital mortality	2 [2–5] 1 (2) 7 0 (0)	5 [3–7] 12 (18) 2 [2, 3] 7 (10)	8 [5–16] 20 (33) 4 [2–6] 12 (20)	**<0.01** **0.01** 0.15 **<0.01**	17 [8–27] 4 [3–9] 7 (39)	16 [7–20] 4 [2–8] 12 (41)	0.48 0.89 0.87

^a^ Cardiac arrest complicated by pulmonary sepsis

^b^ Obstructed airway complicated by pulmonary sepsis

^c^ Acid base disturbance complicated by pulmonary sepsis, ruptured aneurysm complicated by infected endovascular prosthesis, elective orthopedic surgery complicated by infected prosthesis

Information on baseline characteristics, baseline health status, severity on presentation, diagnosis and outcome is compared stratified on the department. Statistical analysis includes one-way ANOVA or *t*-test for normally distributed data, and Kruskal–Wallis test or Mann–Whitney U test for non-normally distributed data. Post hoc comparisons were conducted using the Dunn test and Tukey test with Bonferroni adjustment, when applicable. Normality was assessed using the Anderson–Darling test. Data are presented as mean (SD), n (%), or median [IQR]. Cardiovascular disease (CVD); chronic obstructive pulmonary disease (COPD). Modified from unpublished data by the first author, currently under review.

The ED cohort included patients with sepsis (*n* = 67), SA-AKI (*n* = 61), and non-infectious acutely ill controls (*n* = 65). Sepsis patients were older (median 69 years) than SA-AKI (61) and controls (65; *P* = 0.02), with ~50% female in each group (*P* = 0.94). Sepsis and SA-AKI patients had higher median illness severity scores (NEWS2 scores 7 and 5 vs. 2; *P* < 0.01), higher ICU admission rates (18% and 33% vs. 2%; *P* = 0.01), and higher mortality rates (10% and 20% vs. 0%; *P* = 0.01) compared to controls. The ICU cohort included severe sepsis (*n* = 18) and SA-AKI (*n* = 29) patients needing organ support. SA-AKI patients were older than septic patients (68 vs. 60; *P* = 0.07). Both groups consisted of about ~50% females (*P* = 0.87) and had similar APACHE IV scores (92 vs. 68; *P* = 0.65) and mortality rates (39% vs. 41%; *P* = 0.87).

### Circulating levels of HP, C3a, CHI3L1, and MMP8 are increased in patients with SA-AKI and sepsis

**Fig. 4:**
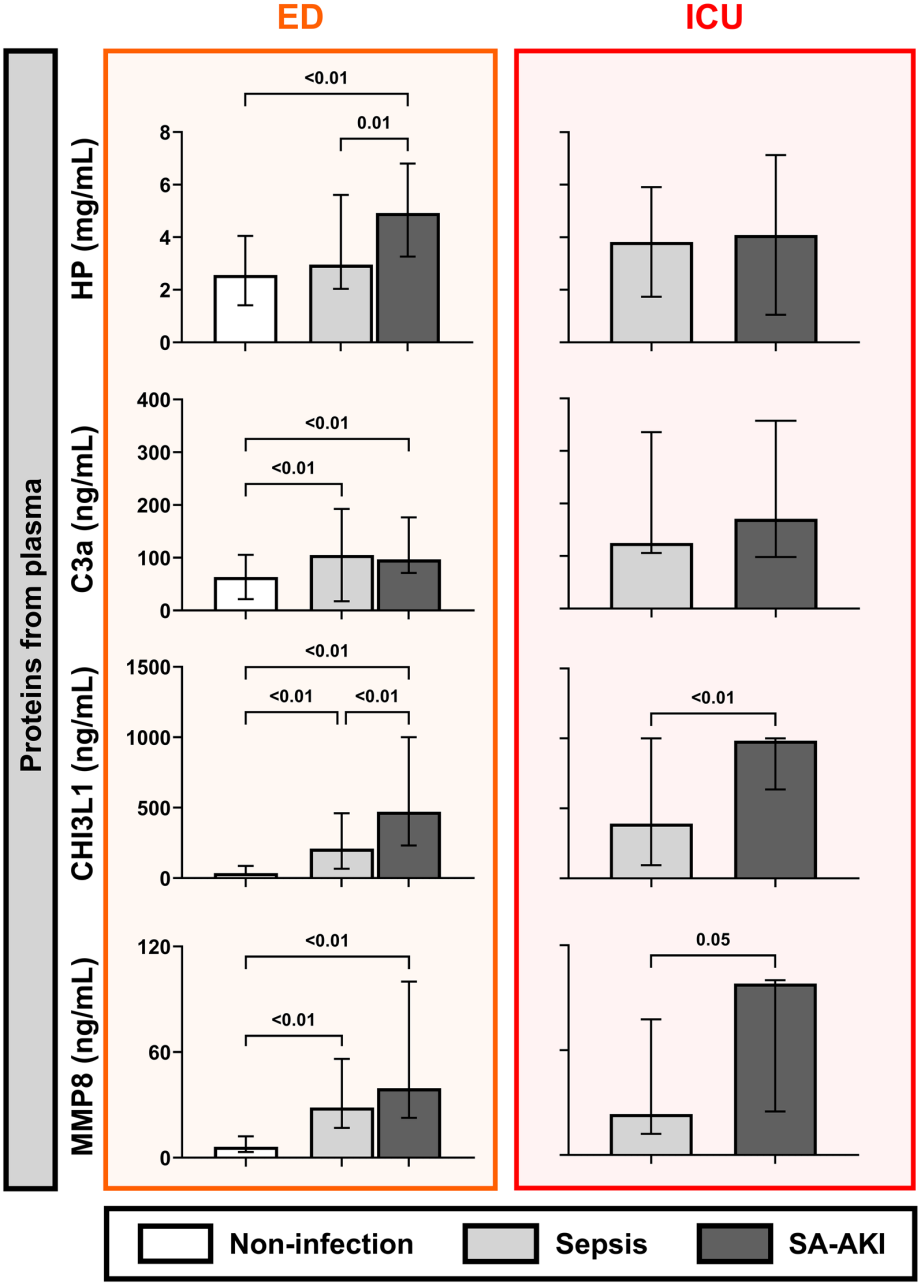
Comparison of selected circulating protein levels in plasma from patients at the emergency department and intensive care unit. Comparison of protein levels across sepsis, SA-AKI, and controls in both an emergency department (ED) cohort (orange), as well as intensive care unit (ICU) patients (red) with more advanced disease. Graphs display column means with SD or median with IQR when applicable. Statistical differences were evaluated using ANOVA or *t*-test for normally distributed data, and Kruskal–Wallis or Mann–Whitney U tests for non-normally distributed data. Statistical significance is indicated in the figure.

We analyzed the plasma levels of the proteins HP, C3a, CHI3L1, and MMP8 (Fig. 4). All proteins were elevated in patients with SA-AKI or sepsis compared to non-infectious acutely ill controls (*P* < 0.01) in the ED cohort. HP (median 3 vs. 5 mg/mL, *P* = 0.01) and CHI3L1 (median 203 vs. 472 ng/mL, *P* < 0.01) levels were higher in SA-AKI patients compared to septic patients. In contrast, circulating C3a and MMP8 did not differ between sepsis and SA-AKI, but were significantly higher when compared to the control group.

In the ICU cohort, which represented more advanced sepsis, levels of CHI3L1 (median 392 vs. 985 ng/mL, *P* < 0.01) and MMP8 (median 23 vs. 98 ng/mL, *P* = 0.05) were significantly higher in patients with SA-AKI compared to those with sepsis. Therefore, all measured proteins showed elevated circulating plasma levels in patients with sepsis or SA-AKI, with CHI3L1 and MMP8 having distinguishably higher levels in SA-AKI compared to sepsis in both early-stage sepsis and advanced-stage sepsis. Overall, the circulating proteins of interest showed limited correlations with clinical parameters of organ (dys)function (Supplemental Figure 2).

## Discussion

In this study, we aimed to characterize EC activation in the context of SA-AKI by identifying molecular markers of the renal microvascular response and assessing their expression across relevant biological models and compartments. Starting from transcriptomic profiling of renal microvascular compartments from CLP-sepsis mice, we selected sepsis-associated candidate microvascular markers for further validation. Using RT-qPCR, we confirmed the upregulation of *HP, C3, Chil3/CHI3L1,* and *MMP8* in renal microvascular compartments in both CLP-sepsis mice and human post-mortem kidney biopsies from patients with SA-AKI. We extended our analysis to plasma and detected parallel increases in protein levels, reinforcing the presence of these markers in the systemic circulation. Importantly, we uncovered that these markers are not kidney-specific; their expression was also elevated in whole lung and liver tissue following sepsis. These findings suggest that these markers could reflect a shared microvascular response to sepsis that occurs in various organs, rather than being confined to the kidney. By targeting the microvascular endothelial niches through LMD, we aimed to capture the endothelial cell response to sepsis while including as little non-vascular signal as possible. These findings therefore represent a strongly enriched endothelial signature and allow a closer look at the endothelial contribution to the observed response. By identifying candidate molecular markers of the endothelial response to sepsis from bench to bedside, we provided a foundation for future efforts to further delineate the microvascular endothelial response in sepsis and to identify associated microvascular biomarkers indicating endothelial dysfunction.

Endothelial dysfunction in sepsis arises from EC activation, which changes barrier integrity, increases leukocyte adhesion, and disrupts local perfusion control [8, 9, 26]. We examined the renal microvasculature to assess endothelial responses within their unique microvascular niches. Upregulation of HP, C3, Chil3/CHI3L1, and MMP8 in renal microvascular compartments of both mice and humans may reflect endothelial activation in sepsis, as supported by prior transcriptomic and proteomic studies. HP, for example, is induced by inflammatory cytokines and acts as a scavenger for cell-free Hb, limiting oxidative stress and EC injury [27–29]. While Toledo et al. observed prominent HP enrichment on renal endothelial surfaces during sepsis [30], its primary sources remain hepatocytes and immune cells in systemic inflammation [31]. The elevated HP levels in our study likely reflect either endothelial surface binding or local immune cell release, positioning HP as a marker of endothelial stress in SA-AKI—even if not directly produced by ECs. Similarly, our study shows that C3, a component of the complement system and has a well-described role in sepsis [32], is consistently upregulated across renal microvascular compartments in both mice and humans, suggesting a conserved role in endothelial activation during SA-AKI. MMP8 promotes endothelial activation by enhancing leukocyte adhesion through upregulation of VCAM-1 and ICAM-1, with its inhibition reducing adhesion molecule expression and inflammatory signaling in human umbilical vein endothelial cells (HUVECs) [33]. In our prior work, we observed neutrophil infiltration in the glomerular compartment during sepsis, and MMP8 is a well-established product of immune cells in inflammatory contexts [34]. Moreover, the temporal and spatial release pattern of *Mmp8* in CLP mice (Fig. 2) parallels that of a marker of infiltrating neutrophils described by Wang et al. Thus, its expression in our study may reflect immune cell-derived release as part of the broader endothelial activation response. *CHI3L1,* on the other hand, seems to have an attenuating effect on the inflammatory response. In a study on aortic EC from patients undergoing coronary artery bypass graft surgery, *CHI3L1* silencing reduced the expression of local proinflammatory mediators [35]. Therefore, HP, C3, Chil3/CHI3L1, and MMP8 have established roles in immune modulation, inflammation, and the oxidative stress response, and our study demonstrates their upregulation as part of the endothelial activation signature in sepsis. Importantly, this upregulation was not limited to the kidney, as we show similar responses in lung and liver tissue, suggesting that these markers reflect a broader, systemic response during sepsis. Therefore, the plasma elevations in our study may be endothelial-derived, but given their role in the inflammatory response, they likely reflect coordinated endothelial-immune interactions. Regardless of their precise cellular source, these findings underscore the translational potential of these markers for monitoring endothelial activation in sepsis.

Interestingly, CHI3L1 was the only protein in our panel that consistently distinguished SA-AKI from sepsis across two patient cohorts. Its association with kidney injury is supported by previous findings where urinary CHI3L1 levels correlated with decreased kidney function and AKI severity [36–38]. While CHI3L1 is predominantly produced by macrophages and neutrophils, recent evidence demonstrates that ECs can also produce CHI3L1 in fibrotic environments [39]. Thus, its upregulation in SA-AKI may reflect a combination of immune cell infiltration and activation as well as local endothelial production. Overall, the consistent ability of CHI3L1 to differentiate sepsis from SA-AKI suggests a specific pathophysiological role in SA-AKI, warranting further investigation into its diagnostic and mechanistic potential.

Our study provides a rational foundation for candidate markers of endothelial activation and dysfunction in sepsis. Recently there has been a growing interest in profiling sepsis based on shared pathophysiological mechanisms, marking a critical step toward precision medicine in sepsis care. Several landmark efforts, including the Sepsis Endotyping in Emergency Care (SENECA) study, the identification of acute respiratory distress syndrome (ARDS) sub-phenotypes from ARDSnet randomized trials, and the Molecular Diagnosis and Risk Stratification of Sepsis (MARS) initiative [10, 12–14], have advanced our understanding of sepsis heterogeneity by delineating distinct clinical and molecular subgroups. However, despite their contributions, these studies have frequently underrepresented the vascular response. For instance, many of the aforementioned studies did not report on relevant vascular biomarkers such as angiopoietin-2 [14], thereby limiting the capacity to assess the role of endothelial dysfunction in driving sub-phenotypic variation. In studies that did include vascular biomarkers, the sub-phenotype classification was largely influenced by dominant inflammatory or immune signals, rendering vascular markers like E-selectin or VCAM-1 indistinguishable across subgroups [12]. Additionally, sub-phenotypes derived through unsupervised hierarchical clustering of global gene expression profiles do not inherently capture mechanistic underpinnings of disease [10]. Without integrating functional data or pathway-level biological context, the resulting sub-phenotypes may lack biological interpretability and fail to highlight specific processes such as the endothelial response. Tangible progress in this area is likely impeded by the incompletely characterized endothelial response, thereby limiting our ability to discern finer distinctions in disease mechanisms. Furthermore, there is currently no clinical marker for endothelial dysfunction, nor an organ-specific endothelial biomarker; as signs like mottling and hypotension may suggest endothelial dysfunction in sepsis, they are not organ- nor vascular bed- specific. Therefore, markers cannot be directly tested against a clinical phenotype, limiting our ability to accurately correlate endothelial responses with specific disease outcomes in sepsis. By adopting an unbiased, biological approach, we identified markers of the EC response to sepsis, laying the groundwork for functional studies to further elucidate their role in endothelial dysfunction and their potential use in sub-phenotyping sepsis.

## Future perspectives

Our study identifies several markers of endothelial inflammatory activation in sepsis, selected based on their induced expression in response to sepsis. These markers hold promise for improving our understanding of the endothelial response during sepsis and may serve as valuable diagnostic or prognostic tools. Future studies should aim to integrate functional endothelial assays alongside comparative marker profiling and longitudinal clinical outcomes to better define and validate the biological relevance of these markers. Such research would provide deeper insights into the mechanisms driving endothelial dysfunction in sepsis. Validation studies involving larger populations are crucial to confirm the clinical utility of these markers. Ultimately, this could contribute to identifying clinically meaningful sub-phenotypes of sepsis, potentially enabling more personalized and targeted treatment strategies. By refining our understanding of sepsis at the microvascular level, these advancements hold potential for improving patient outcomes.

## Limitations

The physiological response of the immune system to sepsis can vary depending on factors such as the patient’s condition, the causative organism, and the site of infection. This variability presents a limitation in all sepsis research. Our study is also constrained by being single-center for both mice and patient studies and therefore lacking external validation. However, it should be noted that our cohort included a large sample size from a diverse hospital setting, encompassing both rural and urban populations, which may enhance the generalizability of our results. LMD enabled isolation of distinct microvascular areas in the kidney during AKI, albeit at the expense of detailed single-cell resolution. As an alternative, flow cytometry (FACS) could have been used to isolate endothelial cells based on surface markers, but this would have resulted in the loss of spatial context as well as transcriptomic changes induced by enzymatic digestion and FACS process [40]. While FACS offers higher cell specificity, LMD was deemed more appropriate for studying endothelial responses in the context of SA-AKI, aligning more closely with our research question. While we initially focused on the kidney, our findings indicate that the uncovered response is not kidney-specific. Therefore, with the benefit of hindsight, it would have been of interest to study the liver and lung microvasculature using LMD, which represents a promising future research avenue. Furthermore, while we deem it likely that the endothelium represents an important source of the increased levels of circulating proteins, we cannot exclude the possibility that these are partially derived from other cell types within the microvascular compartments, or even released systemically in other organs. Our study is limited by the tissues we examined, which restricts our ability to capture the full, whole-body response to sepsis. Moreover, since there is no established definition of endothelial dysfunction, we were unable to directly assess how the identified markers correlate with a clinical reference for endothelial dysfunction in the context of sepsis, which limits our ability to draw precise clinical conclusions. Lastly, we observed a higher proportion of CKD patients in the SA-AKI groups, which may affect the renal microvascular response and warrant further investigation. In summary, while our study provides valuable insights into the microvascular response to sepsis, these limitations should be carefully considered when interpreting the results, particularly regarding their broader applicability to diverse patient populations and clinical settings.

## Conclusion

Our findings reveal markers of the microvascular response to sepsis which include increased levels of *HP, C3, Chil3/CHI3L1,* and *MMP8* both at the transcriptomic level in mouse and human kidney microvasculature and at the protein level in circulating plasma of SA-AKI patients. The upregulation of these markers was shared across multiple organs, and may reflect widespread endothelial activation contributing to sepsis pathophysiology. These markers, as a signature of EC activation, could be further investigated to assess their applicability to aid identification of a vascular sub-phenotype in sepsis.

## Supplementary Information

Additional file 1.

Additional file 2.

Additional file 3.

## Data Availability

All data underlying the figures shown in this manuscript are available in the supplementary files.

## Abbreviations

AKI: Acute kidney injury
CCI: Charlson comorbidity index
CKD: Chronic kidney disease
CLP: Cecal ligation and puncture
ED: Emergency department
ELISA: Enzyme-linked immunosorbent assay
ICU: Intensive care unit
KDIGO: Kidney Disease: Improving Global Outcomes
KIM1: Kidney injury molecule 1
LMD: Laster microdissection
NEWS: National Early Warning Score
NGAL: Neutrophil gelatinase-associated lipocalin
SOFA: Sequential Organ Failure Assessment
SA-AKI: Sepsis associated acute kidney injury
