# Supplementary material for: Uncovering molecular markers of the microvascular endothelial response in sepsis-associated acute kidney injury: a translational study in mice and humans
Source: Intensive Care Med Exp. 2025 Sep 2;13:92. doi: 10.1186/s40635-025-00801-4 (PMC12405097; doi:10.1186/s40635-025-00801-4)
Supplement: Supplementary file 2 — Additional file 2. [file 40635_2025_801_MOESM2_ESM.docx]

**Supplemental file 2.**

**Supplemental material**

**Supplemental Table 1.** Diagnosis at ICU admission

**Supplemental Figure 1**. Altered mRNA transcription levels of kidney damage markers in

microvascular compartments from CLP-induced sepsis in mice.

**Supplemental Figure 2.** Correlation analyses between circulating proteins of interest and clinical parameters of organ function

**Supplemental Table 1**. Diagnosis at ICU admission

Patient diagnoses at ICU admission are presented as absolute numbers and percentages. Five patients initially admitted with a different diagnosis subsequently developed sepsis during their ICU stay.

| Site of infection | ICU  SA-AKI  (N=29)* | ICU  Sepsis  (N=18)* |
| --- | --- | --- |
| Pulmonary tract  Urogenital tract  Abdominal tract  Skin/Soft tissue  Infected prothesis  Other  Unknown  Coinfection | 12 (41)  1 (3)  5 (17)  3 (10)  0  3 (10)  5 (17)  0 | 5 (28)^a^  3 (17)  4 (22)  3 (17)  2 (11)^b^  1 (6)  0  0 |

^a^ Cardiac arrest complicated by pulmonary sepsis, Obstructed airway complicated by pulmonary sepsis, Acid base disturbance complicated by pulmonary sepsis

^b^ Ruptured aneurysm complicated by infected endovascular prothesis, elective orthopaedic surgery complicated by infected prothesis

*Percentages are rounded and therefore do not total exactly 100%.


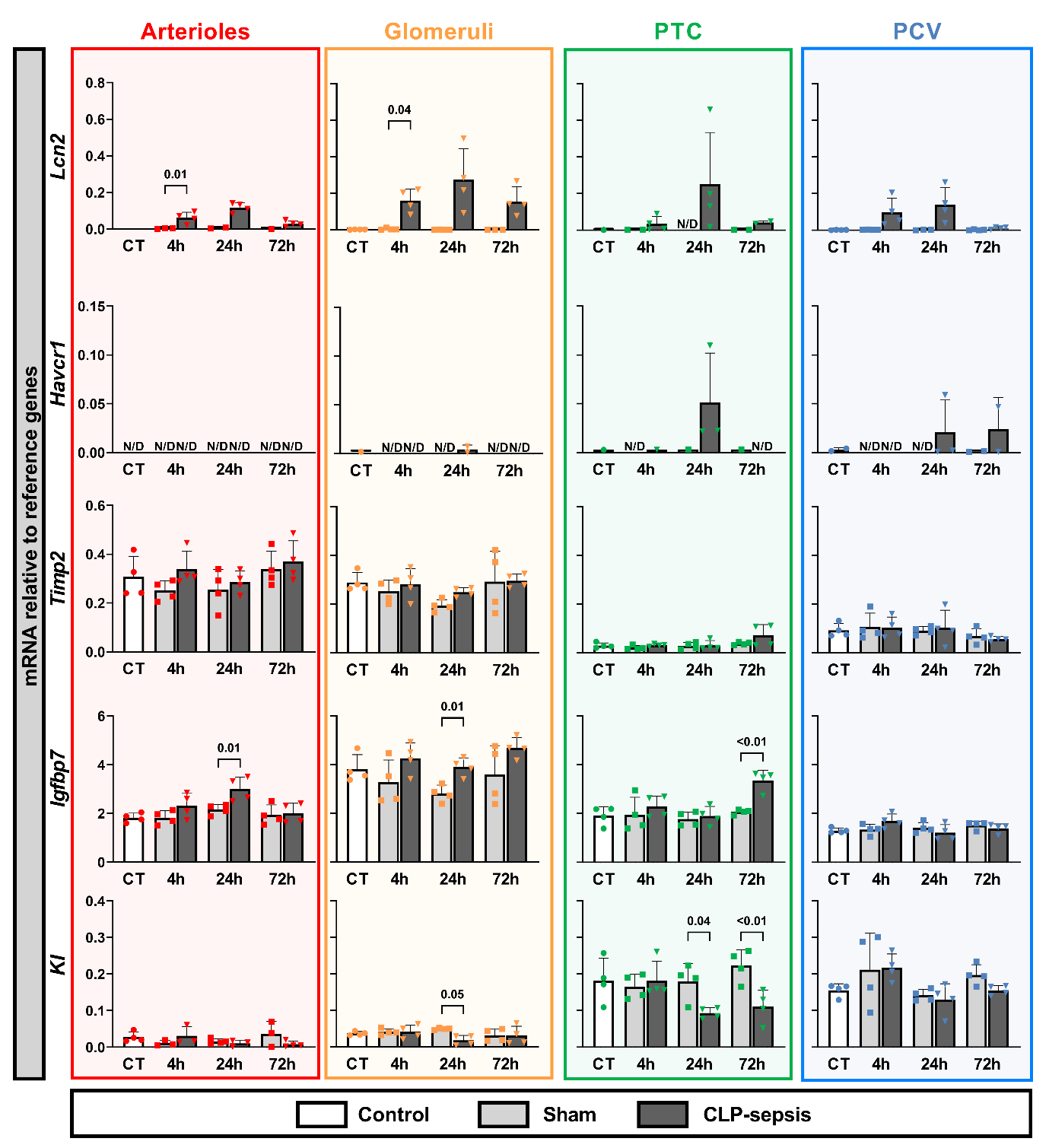


**Supplemental Figure 1.** Altered mRNA transcription levels of kidney damage markers in microvascular compartments from CLP-induced sepsis in mice.

Gene expression levels of Lcn2, Havcr1, Timp2, Igfbp7, and KI were assessed in various microvascular compartments, including arterioles, glomeruli, post tubular capillaries (PTC), and postcapillary venules (PCV). Microvascular compartments were laser micro dissected from mice with CLP-induced sepsis, sham, and control groups. Expression was measured relative to reference genes. Data are presented as mRNA levels. Graphs display column means with SD or median with IQR when applicable. Statistical testing was performed using ANOVA or Welch’s ANOVA with post hoc tests, or Kruskal-Wallis (KW) with post hoc Mann-Whitney U tests where appropriate and when at least three data points were obtained. Statistical significance is indicated when P ≤ 0.05.


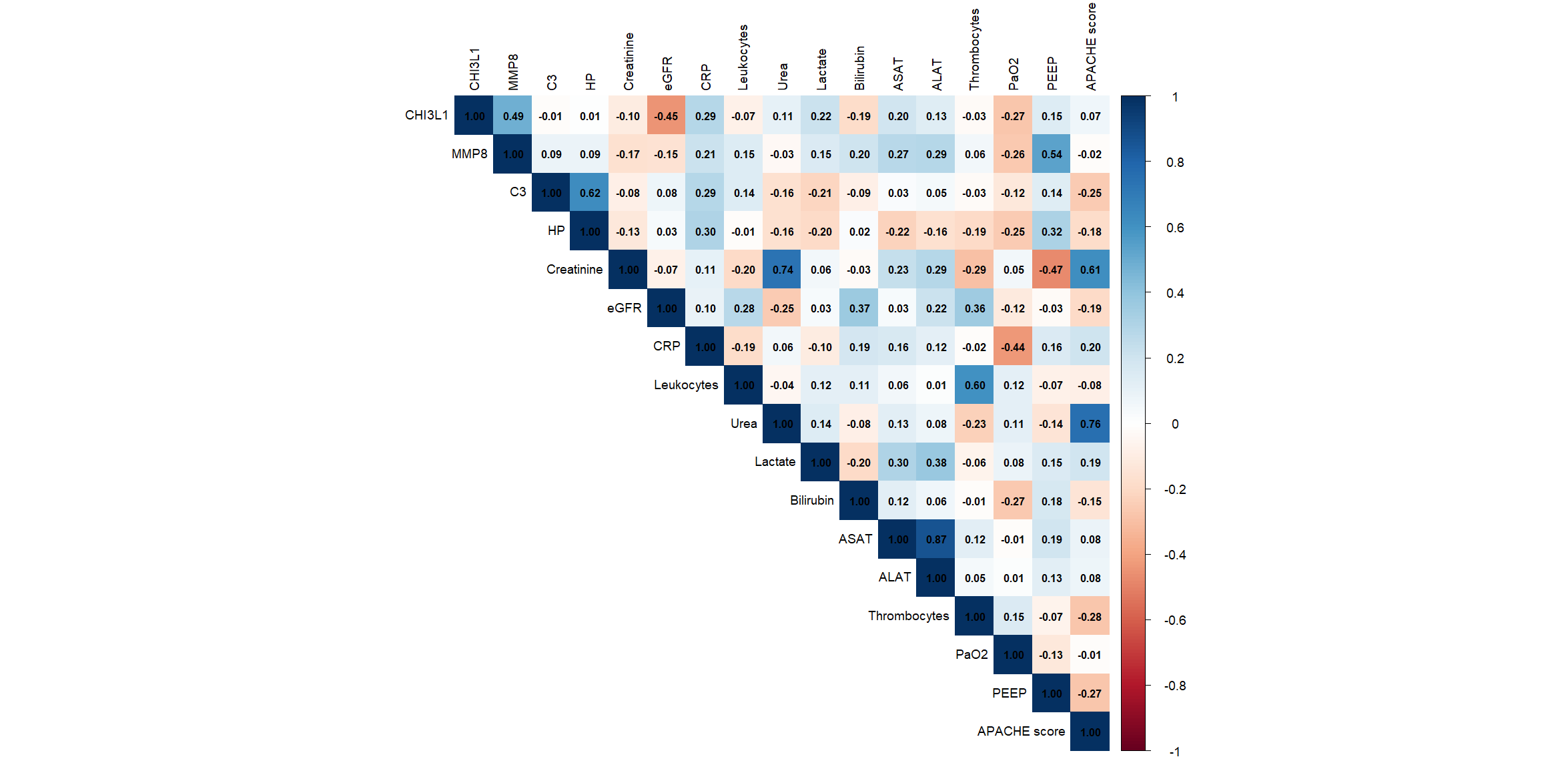

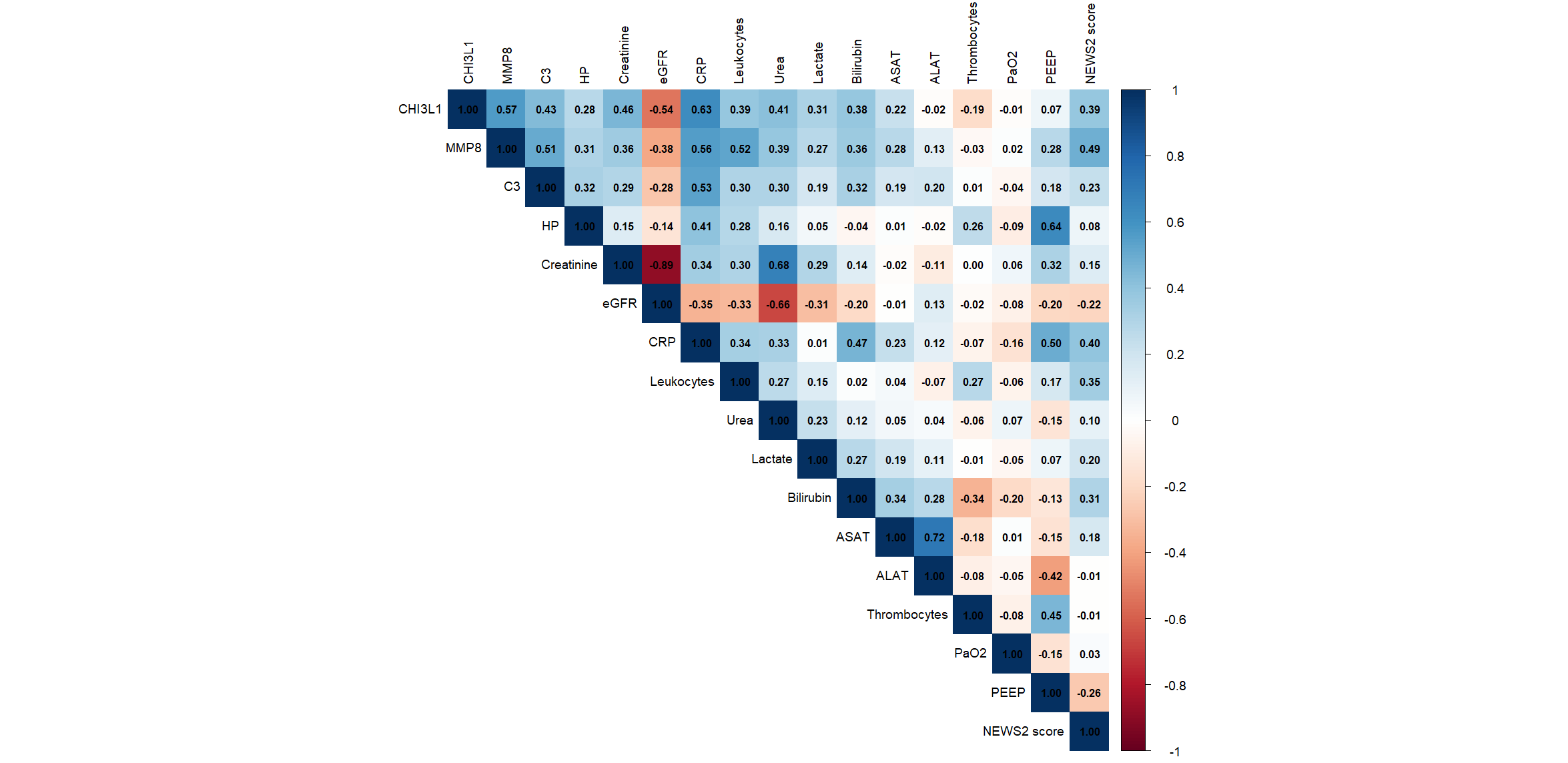


**Supplemental Figure 2. Correlation analyses between circulating proteins of interest and clinical parameters of organ function.** These heatmaps display the correlation coefficients between selected circulating proteins and clinical markers of organ (dys)function of key organs, including liver, kidney, lung, and systemic parameters. The left heatmap represents the emergency department patient cohort, the right heatmap represents the intensive care unit patient cohort. Positive correlations are shown in blue and negative correlations are indicated in red, with the colour intensity corresponding to the strength of the correlation as is indicated by the legend on the right hand side. The exact correlation coefficients are annotated within each cell.
